# Dominant predictors of early post-transplant outcomes based on the Korean Organ Transplantation Registry (KOTRY)

**DOI:** 10.1038/s41598-022-12302-5

**Published:** 2022-05-24

**Authors:** Jong Cheol Jeong, Tai Yeon Koo, Han Ro, Dong Ryeol Lee, Dong Won Lee, Jieun Oh, Jayoun Kim, Dong-Wan Chae, Young Hoon Kim, Kyu Ha Huh, Jae Berm Park, Yeong Hoon Kim, Seungyeup Han, Soo Jin Na Choi, Sik Lee, Sang-Il Min, Jongwon Ha, Myoung Soo Kim, Curie Ahn, Jaeseok Yang, The KOTRY Study Group, Curie Ahn, Myoung Soo Kim, Jaeseok Yang, Jin Min Kong, Oh Jung Kwon, Deok Gie Kim, Cheol Woong Jung, Yeong Hoon Kim, Joong Kyung Kim, Chan-Duck Kim, Ji Won Min, Sung Kwang Park, Yeon Ho Park, Jae Berm Park, Jung Hwan Park, Jong-Won Park, Tae Hyun Ban, Sang Heon Song, Seung Hwan Song, Ho Sik Shin, Chul Woo Yang, Hye Eun Yoon, Kang Wook Lee, Dong Ryeol Lee, Dong Won Lee, Sam Yeol Lee, Sang-Ho Lee, Su Hyung Lee, Yu Ho Lee, Jung Pyo Lee, Jeong-Hoon Lee, Jin Seok Jeon, Heungman Jun, Kyunghwan Jeong, Ku Yong Chung, Hong Rae Cho, Ju Man Ki, Dong-Wan Chae, Soo Jin Na Choi, Sung Shin, Seungyeup Han, Kyu Ha Huh

**Affiliations:** 1 Department of Internal Medicine, Seoul National University Bundang Hospital, Seongnam, Republic of Korea; 2 Department of Nephrology, Seongnam Citizens Medical Center, Seongnam, Republic of Korea; 3 Department of Internal Medicine, Gil Medical Center, Gachon University College of Medicine, Inchon, Republic of Korea; 4 Division of Nephrology, Medicine, Maryknoll Medical Center, Busan, Republic of Korea; 5 Division of Nephrology, Department of Internal Medicine, Pusan National University School of Medicine, Busan, Republic of Korea; 6 Department of Internal Medicine, Kangdong Sacred Heart Hospital, Hallym University College of Medicine, Seoul, Republic of Korea; 7 Medical Research Collaborating Center, Seoul National University Hospital, Seoul National University College of Medicine, Seoul, Republic of Korea; 8 Department of Internal Medicine, Seoul National University College of Medicine, Seoul, Republic of Korea; 9 Department of Surgery, Asan Medical Center, University of Ulsan College of Medicine, Seoul, Republic of Korea; 10 Department of Surgery, Yonsei University College of Medicine, Seoul, Republic of Korea; 11 Department of Surgery, Samsung Medical Center, Sungkyunkwan University School of Medicine, Seoul, Republic of Korea; 12 Department of Internal Medicine, Inje University Busan Paik Hospital, Busan, Republic of Korea; 13 Department of Internal Medicine, Keimyung University School of Medicine, Daegu, Republic of Korea; 14 Department of Surgery, Chonnam National University Medical School, Gwangju, Republic of Korea; 15 Department of Internal Medicine, Jeonbuk National University Medical School, Jeonju, Republic of Korea; 16 Department of Surgery, Seoul National University Hospital, Seoul National University College of Medicine, Seoul, Republic of Korea; 17 Transplantation Research Institute, Medical Research Center, Seoul National University College of Medicine, Seoul, Republic of Korea; 18 Korean Organ Transplantation Registry Foundation, 3rd Floor, Myeongryun-gil 22, Jongno-gu, Seoul, Republic of Korea; 19 Laboratory of Transplantation Immunology, Division of Nephrology, Department of Internal Medicine, Severance Hospital, Yonsei University College of Medicine, Room 336, 3rd Floor, 50 Yonsei-ro, Seodaemun-gu, Seoul, 03722 Republic of Korea; 20 Department of Nephrology, BHS Hanseo Hospital, Busan, Republic of Korea; 21 Department of Surgery, College of Medicine, Han Yang University, Seoul, Republic of Korea; 22 Department of Surgery, Yonsei University Wonju College of Medicine, Wonju Severance Christian Hospital, Wonju, Republic of Korea; 23 Department of Surgery, Korea University Anam Hospital, Seoul, Republic of Korea; 24 Department of Internal Medicine, Bongseng Memorial Hospital, Busan, Republic of Korea; 25 Department of Internal Medicine, School of Medicine, Kyungpook National University Hospital, Daegu, Republic of Korea; 26 Division of Nephrology, Department of Internal Medicine, Bucheon St. Mary’s Hospital, Bucheon-si, Republic of Korea; 27 Department of Surgery, Gil Medical Center, Gachon University College of Medicine, Incheon, Republic of Korea; 28 Department of Nephrology, Konkuk University School of Medicine, Seoul, Republic of Korea; 29 Department of Nephrology, Yeungnam University Hospital, Daegu, Republic of Korea; 30 Division of Nephrology, Department of Internal Medicine, Eunpyeong St. Mary’s Hospital, Seoul, Republic of Korea; 31 Department of Surgery, Ewha Womans University Seoul Hospital, Seoul, Republic of Korea; 32 Division of Nephrology, Department of Internal Medicine, Kosin University College of Medicine, Busan, Republic of Korea; 33 Division of Nephrology, Department of Internal Medicine, Seoul St. Mary’s Hospital, Seoul, Republic of Korea; 34 Department of Internal Medicine, Incheon St. Mary’s Hospital, College of Medicine, The Catholic University of Korea College of Medicine, Seoul, Republic of Korea; 35 Department of Nephrology, Chungnam National University Hospital, Daejeon, Republic of Korea; 36 Department of Surgery, Kangdong Sacred Heart Hospital, Hallym University College of Medicine, Chuncheon, Republic of Korea; 37 Department of Nephrology, Kyung Hee University Hospital at Gangdong, Seoul, Republic of Korea; 38 Department of Surgery, Ajou University School of Medicine, Suwon, Republic of Korea; 39 Division of Nephrology, Department of Internal Medicine, CHA Bundang Medical Center, CHA University, Seongnam, Korea; 40 Department of Nephrology, SMG-SNU Boramae Medical Center, Seoul, Republic of Korea; 41 Department of Surgery, Myongji Hospital, Goyang, Republic of Korea; 42 Department of Internal Medicine, Soonchunhyang University Seoul Hospital, Seoul, Republic of Korea; 43 Department of Surgery, Inje University Ilsan Paik Hospital, Goyang, Republic of Korea; 44 Department of Internal Medicine, Kyung Hee University College of Medicine, Seoul, Republic of Korea; 45 Department of Surgery, Ewha Womans University Mokdong Hospital, Seoul, Republic of Korea; 46 Department of Surgery, Ulsan University Hospital, Ulsan, Republic of Korea; 47 Department of Surgery, Gangnam Severance Hospital, Yonsei University College of Medicine, Seoul, Republic of Korea

**Keywords:** Allotransplantation, Epidemiology

## Abstract

Data for Asian kidney transplants are very limited. We investigated the relative importance of prognostic markers in Asian kidney transplants by using Korean Organ Transplantation Registry (KOTRY) cohort. Prediction models were developed by data-driven variable selection approach. The relative importance of the selected predictors was measured by dominance analysis. A total of 4854 kidney transplant donor-recipient pairs were analyzed. Overall patient survival rates were 99.8%, 98.8%, and 91.8% at 1, 3, and 5 years, respectively. Death-censored graft survival rates were 98.4%, 97.0%, and 95.8% at 1, 3, and 5 years. Biopsy-proven acute rejection free survival rates were 90.1%, 87.4%, and 87.03% at 1, 3, and 5 years. The top 3 dominant predictors for recipient mortality within 1 year were recipient cardiovascular disease history, deceased donor, and recipient age. The dominant predictors for death-censored graft loss within 1 year were acute rejection, deceased donor, and desensitization. The dominant predictors to acute rejection within 1 year were donor age, HLA mismatched numbers, and desensitization. We presented clinical characteristics of patients enrolled in KOTRY during the last 5 years and investigated dominant predictors for early post-transplant outcomes, which would be useful for clinical decision-making based on quantitative measures.

## Introduction

The first kidney transplant in South Korea was conducted in 1969, and the procedure was popularized in the 1990s. The number of kidney transplants has been increasing since the 1980s. In past eras, most kidney transplant programs were based on living donations, and the deceased donor kidney transplant program operated in some centers^1^. After the introduction of brain death legislation and the establishment of regulatory agencies, deceased donor kidney transplant programs showed a decline in the early 2000s. However, with the effort to promote deceased organ donation and transparent allocation, deceased donor kidney transplants have rapidly increased, accounting for about 45% of total kidney transplants in recent years^2–4^. Accompanying this expansion of transplant volume, electronic claim databases became good resources for transplant research in South Korea, from which several good reports were produced overviewing Korean kidney transplants^5,6^. However, data based on claim reports or administrative databases generally lack many clinical details, and they should be supplemented by observational cohort or registry data.

In the transplant field, nationwide and international transplant registries have provided many valuable data resources and led to the development of a clinical science of transplantation^7–9^. The Korean Organ Transplantation Registry (KOTRY) has operated as an observational cohort of organ transplantation since 2012. In 2014, we reported the first nationwide retrospective data summary of 4,500 kidney transplant cases that had been performed from 2009 to 2012^10^. Based on that project, a prospective observational cohort involving five different organ transplants (kidney, liver, heart, lung, and pancreas) started in 2014 under the same name (KOTRY)^11^. We provide a data summary of 5 years of enrollments of kidney transplant donor-recipient pairs in this paper. In addition, we investigated major dominant predictors of early kidney transplant outcomes including survival of patients and grafts, occurrence of acute rejection, and estimated glomerular filtration rate (eGFR) of transplanted grafts.

In terms of statistical modelling, most clinical epidemiological studies have used inferential methods based on the knowledge of experts and predefined hypotheses. On the contrary, the data-driven approach does not depend on prior hypotheses, which are usually used to build a prediction or prognostic model. Prognostic models in kidney transplants are an active area of research; however, the studies were scarce that compared the relative importance or the relative weight of clinical predictors for post-transplant outcomes^12–23^. In the present study, we compared the relative importance of clinical predictors based on a data-driven approach in addition to the 5 years outcome reporting of KOTRY.

## Results

### Descriptive baseline characteristics of Korean Organ Transplantation Registry (KOTRY)

In Table 1, baseline characteristics of kidney transplant recipients are described. Mean age of the recipients was 49.1 ± 11.5 years. In deceased donor kidney transplants, the mean age of recipients was higher (51.7 ± 10.6, *p* < 0.001). Females accounted for 40.6% of recipients. More male recipients received deceased donor kidneys. Mean body mass index (BMI) was 23.1 ± 3.6 kg/m^2^; mean systolic blood pressure before kidney transplant was 139.2 ± 20.8 mmHg. The proportion of current smokers was 8.6%. As comorbidities, diabetes were present in 29.8% and hypertension in 89.7% of recipients. The proportion of cardiovascular disease was 6.1%, which was higher in deceased donor kidney transplant recipients. History of malignancy was present in 6.6%. The most common cause of end-stage renal disease (ESRD) was chronic glomerulonephritis (33.3%), followed by diabetic nephropathy (23.5%). Hemodialysis was the most frequently used dialysis modality before transplant (70.9%). Preemptive transplantation was performed in 24.0% of living donor kidney transplants. Mean waiting time for deceased donor KT was 68.7 ± 38.0 months. Re-transplantation was done in 7.8% of cases. Mean number of HLA mismatches was 3.4 ± 1.8. Mean panel reactive antibody positivity percentage was 11.7 ± 24.3 in class I and 11.7 ± 24.8 in class II. As an induction agent, Basiliximab was used in 80.6% of kidney transplants, and ATG was used in 31.8% of deceased donor kidney transplants. Tacrolimus was the main calcineurin inhibitor (96.2%). Early steroid withdrawal was done in 2.0% of patients. Donor data was described as cases (Table 2). Mean age of donor cases was 47.2 ± 12.7 years. Females were more prevalent in living donors, and males were more prevalent in deceased donors. Diabetic donors accounted for 12.0% of deceased donors and 1.2% of living donors. Donors with hypertension accounted for 24.5% of deceased donor kidney transplants and 9.5% of living donor kidney transplants. Mean BMI of donors was 23.8 ± 3.4 kg/m^2^, and mean pre-transplant SBP of donors was 122.4 ± 17.2 mmHg. Proportion of smokers was 17.3% in living donors. Mean cold ischemic time was 289.0 ± 134.5 mins in deceased donors. Continuous renal replacement therapy was applied to 6.1% of deceased donors. Extracorporeal membrane oxygenator was applied to 2.5% of deceased donors.

**Table 1:** Baseline clinical characteristics of the kidney transplant recipients of Korean Organ Transplantation Registry (2014–2018).

Variables	Total (n = 4854)	Living (n = 3050)	Deceased (n = 1804)	*p*
Age, yrs	49.1 ± 11.5	47.6 ± 11.7	51.7 ± 10.6	< 0.001
Female sex	1969 (40.6)	1268 (41.6)	701 (38.9)	0.063
Body mass index, kg/m^2^	23.1 ± 3.6	23.2 ± 3.7	23.0 ± 3.3	0.189
SBP, mmHg	139.2 ± 20.8	136.0 ± 19.1	144.7 ± 22.3	< 0.001
DBP, mmHg	83.7 ± 12.8	83.2 ± 12.5	84.5 ± 13.3	0.001
**Smoking**				< 0.001
Never	3679 (75.8)	2294 (75.2)	1385 (76.8)	
Current	416 (8.6)	236 (7.7)	180 (10.0)	
Former	705 (14.5)	493 (16.2)	212 (11.8)	
Unknown	54 (1.1)	27 (0.9)	27 (1.5)	
**Comorbidities**				
Diabetes	1449 (29.8)	920 (30.0)	529 (29.4)	0.536
Hypertension	4373 (89.7)	2750 (89.7)	1623 (89.6)	0.957
Cardiovascular disease	532 (11.0)	271 (8.9)	261 (14.5)	< 0.001
Ischemic heart disease	344 (7.1)	184 (6.0)	160 (8.9)	
Heart failure	103 (2.1)	43 (1.4)	60 (3.3)	
Arrhythmia	73 (1.5)	27(0.9)	46 (2.5)	
Valvular heart disease	18 (0.4)	9 (0.3)	9 (0.5)	
Other cardiovascular disease	29 (0.6)	19 (0.6)	10 (0.6)	
Malignancies	320 (6.6)	185 (6.0)	135 (7.4)	0.055
**Cause of end stage renal disease**				< 0.001
Diabetic nephropathy	1140 (23.5)	713 (23.3)	427 (23.7)	
Hypertension	762 (15.7)	412 (13.5)	350 (19.4)	
Glomerulonephritis	1615 (33.3)	1070 (35.1)	545 (30.2)	
ADPKD	233 (4.8)	150 (4.9)	83 (4.6)	
Other	150 (3.1)	95 (3.1)	55 (3.0)	
Unknown	954 (19.7)	610 (20.0)	344 (19.1)	
**Dialysis before transplantation**				< 0.001
Hemodialysis	3443 (70.9)	2018 (66.2)	1425 (79.0)	
Peritoneal dialysis	618 (12.7)	241 (7.9)	377 (20.9)	
Kidney transplant	59 (1.2)	59 (1.9)	0 (0)	
Preemptive	734 (15.1)	732 (24.0)	2 (0.1)	
Duration of waitlist, mos	57.0 ± 42.4	8.6 ± 18.5	68.7 ± 38.0	< 0.001
Repeated kidney transplantation	377 (7.8)	217 (7.1)	160 (8.9)	0.137
Desensitization	1103 (22.7)	1061 (34.8)	42 (2.3)	< 0.001
HLA mismatch numbers (Class I)	2.3 ± 1.2	2.2 ± 1.2	2.3 ± 1.3	< 0.001
HLA mismatch numbers (Class II)	1.1 ± 0.7	1.1 ± 0.7	1.1 ± 0.8	0.800
Panel reactive antibodies (Class I), %	11.7 ± 24.3	10.4 ± 22.5	13.8 ± 27.0	< 0.001
Panel reactive antibodies (Class II), %	11.7 ± 24.8	10.4 ± 23.0	13.9 ± 27.4	< 0.001
**Induction agent**				< 0.001
Anti-thymocyte globulin	1009 (20.9)	435 (14.3)	574 (31.8)	
Basiliximab	3911 (80.6)	2628 (86.2)	1283 (71.1)	
No induction	49 (1.0)	27 (0.9)	22 (1.2)	
**Calcineurin inhibitor**				< 0.001
Tacrolimus	4671 (96.2)	2901 (95.1)	1770 (98.1)	
Cyclosporin A	154 (3.2)	133 (4.4)	21 (1.2)	
No Calcineurin inhibitors	55 (1.1)	35 (1.1)	20 (1.1)	
Mycophenolic mofetil	4205 (86.6)	2644 (86.7)	1561 (86.5)	0.875
mTOR inhibitor	51 (1.1)	36 (1.2)	15 (0.8)	0.249
**Steroid**				0.194
Yes	4759 (98.0)	2996 (98.2)	1763 (97.7)	
No	95 (2.0)	54 (1.8)	41 (2.3)	

ADPKD, autosomal dominant polycystic kidney disease; DBP, diastolic blood pressure; HLA, human leukocyte antigen; SBP, systolic blood pressure.

Data are presented as number (%) or mean ± standard deviation.

**Table 2:** Baseline clinical characteristics of the kidney transplant donors of Korean Organ Transplantation Registry (2014–2018).

Variables	Total (n = 4854)	Living (n = 3050)	Deceased (n = 1804)	*p*
Age, yrs	47.2 ± 12.7	46.2 ± 11.7	49.0 ± 14.0	< 0.001
Female sex	2253 (46.4)	1716 (56.3)	537 (29.8)	< 0.001
**Comorbidities**				
Diabetes	252 (5.2)	36 (1.2)	216 (12.0)	< 0.001
Hypertension	733 (15.1)	291 (9.5)	442 (24.5)	< 0.001
Body mass index, kg/m^2^	23.8 ± 3.4	24.2 ± 3.2	23.2 ± 3.7	< 0.001
SBP, mmHg	122.4 ± 17.2	122.2 ± 13.9	122.7 ± 21.9	0.418
DBP, mmHg	75.3 ± 12.7	76.3 ± 10.0	73.6 ± 16.2	< 0.001
**Smoking**				< 0.001
Never	3112 (64.1)	2233 (73.2)	879 (48.7)	
Current	1210 (24.9)	531 (17.4)	679 (37.6)	
Former	314 (6.5)	244 (8.0)	70 (3.9)	
Unknown	218 (4.5)	42 (1.4)	176 (9.8)	
Cold ischemic time, mins	125.9 ± 137.4	52.8 ± 44.3	289.0 ± 134.5	< 0.001
CRRT	110 (2.3)	0	110 (6.1)	
ECMO	45 (0.9)	0	45 (2.5)	

CRRT, continuous renal replacement therapy; DBP, diastolic blood pressure; ECMO, extracorporeal membrane oxygenation; SBP, systolic blood pressure.

### Patient survival and cause of death

**Figure 1:**
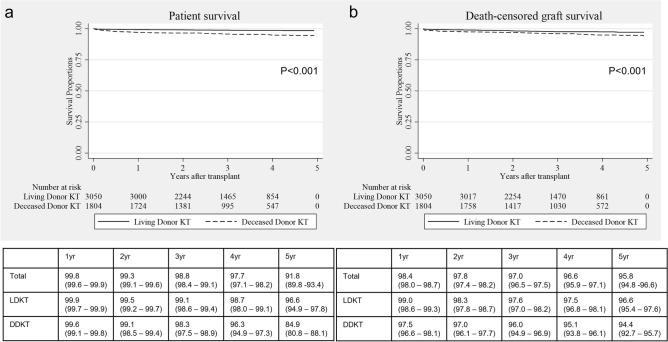
Patient (**a**) and death-censored graft (**b**) survival of Korean Organ Transplantation Registry.

Overall patient survival rates were 99.8%, 98.8%, and 91.8% at 1, 3, and 5 years, respectively. Among living donor kidney transplant recipients, patient survival rates at 1, 3, and 5 years were 99.9%, 99.1%, and 96.6%, respectively. Among deceased donor kidney transplants, patient survival rates at 1, 3, and 5 years were 99.6%, 98.3%, 84.9%, respectively (Fig. 1a). The most common causes of death were infection (45.0%), followed by cardiovascular disease (10.0%), the latter occurring exclusively in deceased donor kidney transplants (Supplementary Table 1). Infection as the cause of death was defined regardless of microorganism.

### Death-censored graft survival and cause of graft failure

Death-censored graft survival rates were 98.4%, 97.0%, and 95.8% at 1, 3, and 5 years, respectively. Among living donor kidney transplant recipients, death-censored graft survival rates were 99.0%, 97.6%, and 96.6% at 1, 3, and 5 years, respectively. Among deceased donor kidney transplants, death-censored graft survival rates were 97.5%, 96.0%, and 94.4% at 1, 3, and 5 years, respectively (Fig. 1b). Rejection (43.5%) was the most common cause of graft loss. Primary graft failure occurred in 11.1% of graft failures. BK virus nephropathy was the third most common cause of graft loss (5.6%) (Supplementary Table 2).

### Indication of kidney biopsy and pathology outcomes

**Figure 2:**
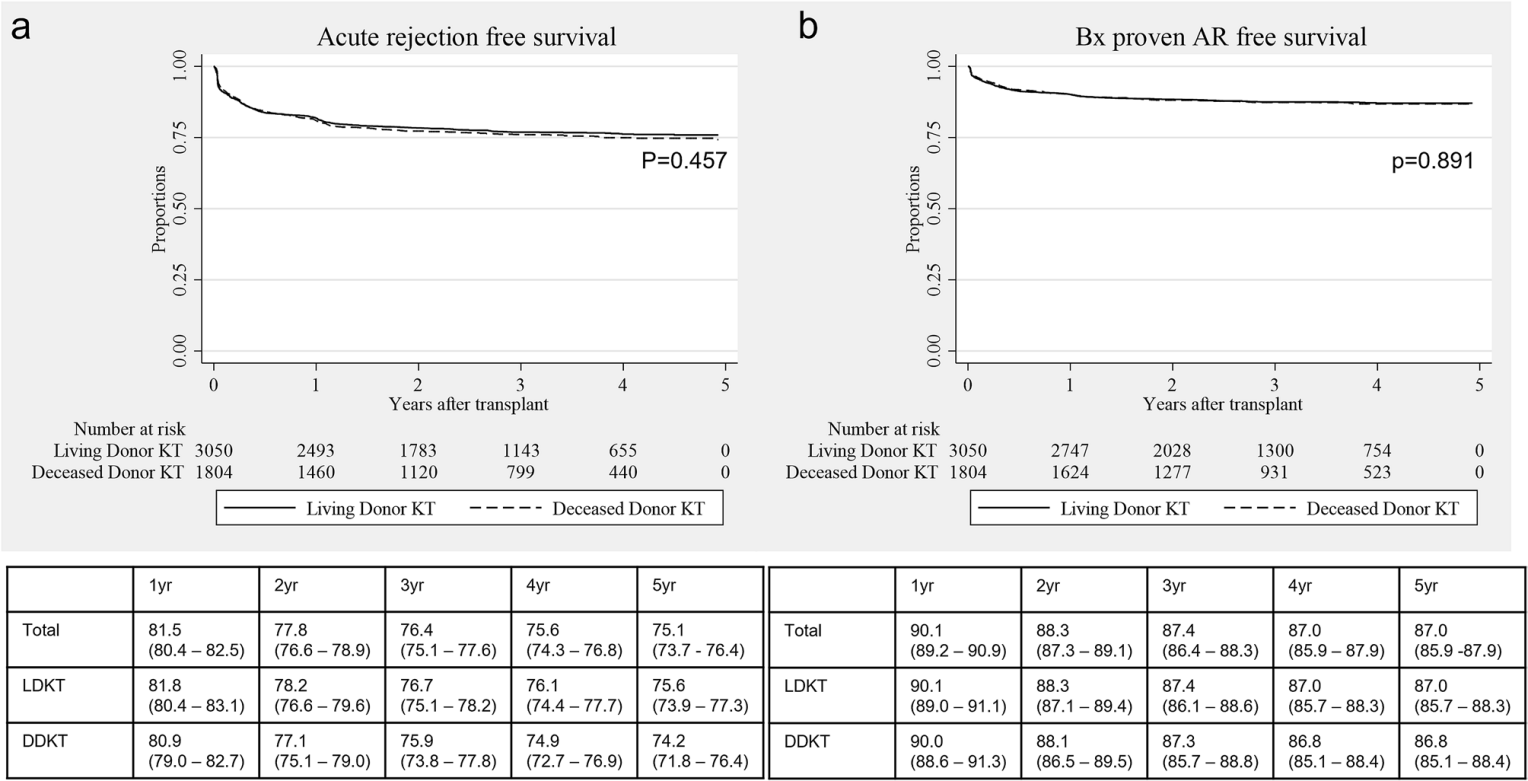
Acute rejection free- (**a**) and biopsy-proven acute rejection free- (**b**) survival of Korean Organ Transplantation Registry.

A total of 3,712 kidney biopsies were performed. Among them, 58.6% were protocol biopsies. The most common indication of for-cause biopsy was increased creatinine (37.2%) (Supplementary Table 3). Among for-cause biopsies, acute T-cell mediated rejection accounted for 25.6%, acute antibody mediated rejection for 13.9%, and borderline change for 21.1%. Recurrent glomerulonephritis occurred in 9.7% of cases and BK-virus-associated nephropathy in 7.9%. If protocol biopsies are included, the proportion of biopsy findings declined; however, the proportion of borderline change was similar (Table 3). Acute rejection free survival rates were 81.5%, 76.4%, and 75.1% at 1, 3, and 5 years, respectively. Among living donor kidney transplant recipients, acute rejection free survival rates were 81.8%, 76.7%, and 75.6% at 1, 3, and 5 years, respectively. Among deceased donor kidney transplants, acute rejection free survival rates were 80.9%, 75.9%, and 74.2% at 1, 3, and 5 years, respectively (Fig. 2a). Biopsy-proven acute rejection free survival rates were 90.1%, 87.4%, and 87.0% at 1, 3, and 5 years, respectively. Among living donor kidney transplant recipients, biopsy-proven acute rejection free survival rates were 90.1%, 87.4%, and 87.0% at 1, 3, and 5 years, respectively. Among deceased donor kidney transplant recipients, biopsy-proven acute rejection free survival rates were 90.0%, 87.3%, and 86.8% at 1, 3, and 5 years, respectively (Fig. 2b).

**Table 3:** Result of post-transplant kidney allograft biopsy.

Variables	Including protocol biopsies			Only for-cause biopsies		
	Total (n = 3712)	Living (n = 2267)	Deceased (n = 1445)	Total (n = 1538)	Living (n = 869)	Deceased (n = 669)
Borderline change	723 (19.5%)	444 (19.6%)	279 (19.3%)	325 (21.1%)	182 (20.9%)	143 (21.4%)
Acute T-cell mediated rejection	549 (14.8%)	341 (15.0%)	208 (14.4%)	394 (25.6%)	247 (28.4%)	147 (22.0%)
Acute antibody mediated rejection	263 (7.1%)	163 (7.2%)	100 (6.9%)	214 (13.9%)	126 (14.5%)	88 (13.2%)
Chronic active T cell mediated rejection	48 (1.3%)	26 (1.2%)	22 (1.5%)	45 (2.9%)	24 (2.8%)	21 (3.1%)
Chronic active antibody mediated rejection	46 (1.2%)	27 (1.2%)	19 (1.3%)	40 (2.6%)	22 (2.5%)	18 (2.7%)
Interstitial fibrosis and tubular atrophy	526 (14.2%)	277 (12.2%)	249 (17.2%)	268 (17.4%)	140 (16.1%)	128 (19.1%)
BK nephropathy	134 (3.6%)	70 (3.1%)	64 (4.4%)	121 (7.9%)	62 (7.1%)	59 (8.8%)
Glomerulonephritis	206 (5.6%)	110 (4.9%)	96 (6.6%)	149 (9.7%)	82 (9.4%)	67 (10.1%)
Calcineurin inhibitor toxicity	192 (5.2%)	95 (4.2%)	97 (6.7%)	108 (7.0%)	58 (6.7%)	50 (7.5%)
Others	765 (20.6%)	423 (18.7%)	342 (23.7%)	410 (26.7%)	246 (28.3%)	164 (24.5%)

Multiple selections are allowed.

### Dominant predictors for patient survival

To explore predictors for patient survival, we applied cross-validated LASSO, which resulted in all entered variables being selected at the optimum lambda. We interpret this due to sufficient n to predictors (not p > n condition), where LASSO might not show its strength in variable selection (Supplementary Table 4). Traditional backward stepwise selection showed reduced predictors from 19 to 11 variables at the threshold of p-value under 0.20 (Supplementary Table 5 and Table 4). To compare the relative importance of predictors, we chose 1-year patient survival as outcome and applied the dominance analysis method to the chosen predictors in previous backward stepwise selection. Cardiovascular disease history of recipient was the most dominant predictor for 1-year patient death, followed by deceased donor kidney transplant, recipient age, duration of dialysis, and diabetes of recipient (Table 4). Among cardiovascular disease history of recipients, all subcategories of cardiovascular disease except for arrhythmia history were significant predictors to post-transplant patient 1 year survival (Supplementary Table 6).

**Table 4:** Selected predictors to 1 year patient death and variable rank by dominance.

Variables	Odds ratios (95% C.I.)	Beta	Standardized beta	*p*	Rank
Cardiovascular disease (recipients)	3.110 (1.852–5.222)	1.135	0.352	< 0.001	1
Deceased donor	2.896 (1.442–5.776)	1.060	0.505	0.003	2
Age (recipients)	1.044 (1.016–1.073)	0.043	0.497	0.002	3
Duration of renal replacement therapy, months	1.006 (1.002–1.010)	0.006	0.362	0.001	4
Diabetes (recipients)	1.423 (0.838–2.414)	0.352	0.161	0.191	5
Diabetes (donors)	1.711 (0.866–3.380)	0.537	0.120	0.122	6
HLA mismatch numbers	1.139 (0.985–1.318)	0.130	0.229	0.079	8
Body mass index (recipients), kg/m^2^	1.071 (0.985–1.149)	0.068	0.245	0.056	7
Body mass index (donors), kg/m^2^	0.927 (0.864–0.995)	− 0.076	− 0.255	0.035	9
Desensitization	2.484 (1.209–5.104)	0.910	0.385	0.013	10
Female sex	0.693 (0.406–1.185)	− 0.366	− 0.180	0.181	11

HLA, human leukocyte antigen.

### Dominant predictors for graft survival, post-transplant eGFR, and acute rejection

**Figure 3:**
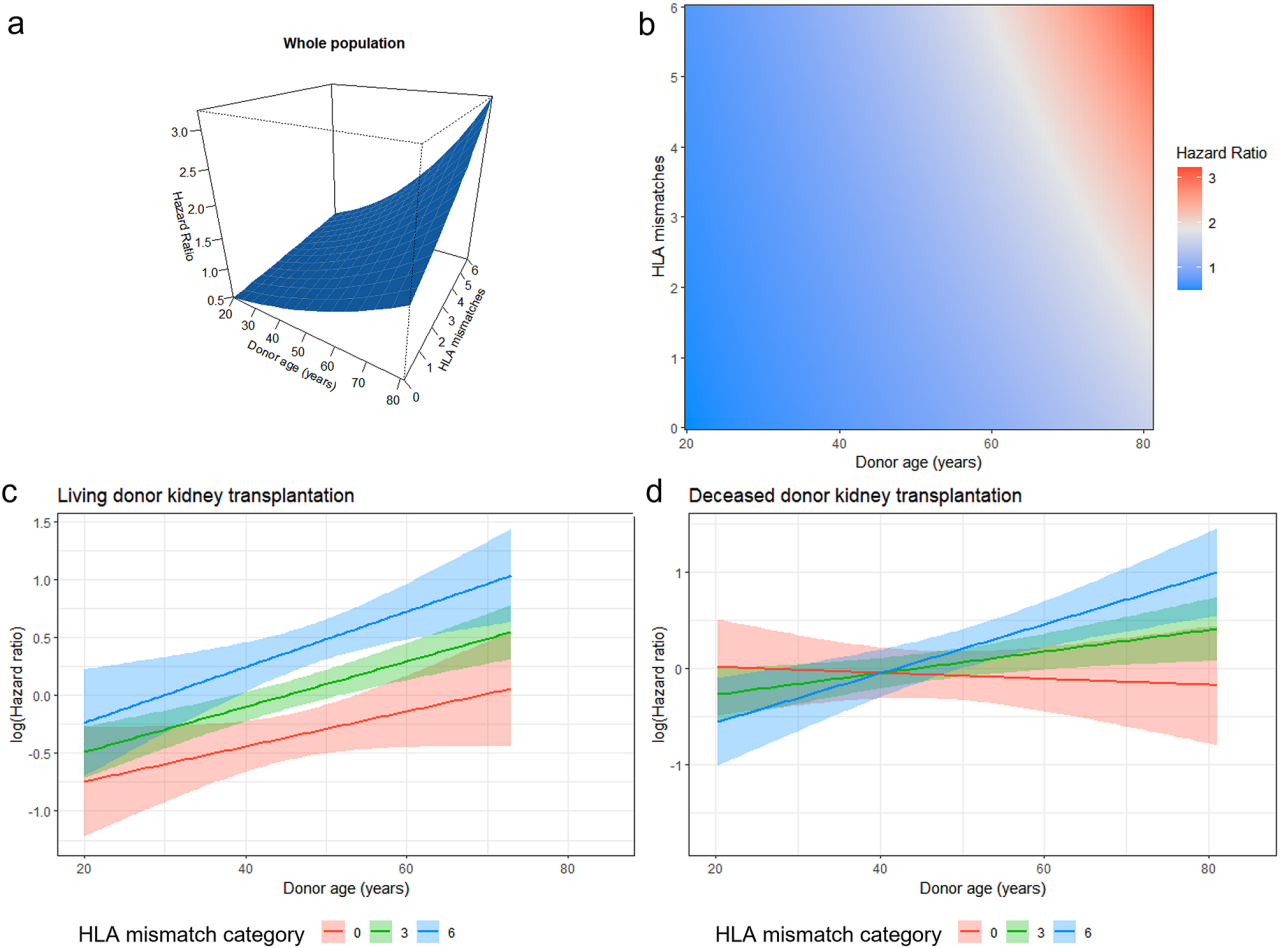
Hazard ratios of donor age to post-transplantation acute rejection within 1 year. (**a**) Three dimensional visualization of hazard ratio of donor age according to HLA mismatch numbers in overall study population (**b**) Two dimensional contour map of the hazard ratio of donor age according to HLA mismatch numbers in overall study population (**c**) Stratified hazard ratio of donor age in living donor kidney transplantation subpopulation (**d**) Stratified hazard ratio of donor age in deceased donor kidney transplantation subpopulation. Red line indicates logarithm of hazard ratio of donor age in HLA full match group. Each colored area indicates its 95% confidence interval. Green line indicates logarithm of hazard ratio of donor age in moderate HLA mismatch group (as representative, hazard ratio line of HLA mismatch number 3 is used). Blue line indicates logarithm of hazard ratio of donor age in high HLA mismatch group (hazard ratio line of HLA mismatch number 6 is used). All graphs are the results of multivariable regression analyses which included donor age, HLA mismatch numbers, desensitization, recipient sex, recipient age, donor hypertension, recipient blood pressure, deceased donor, duration of renal replacement therapy, ever smoking in recipients.

**Figure 4:**
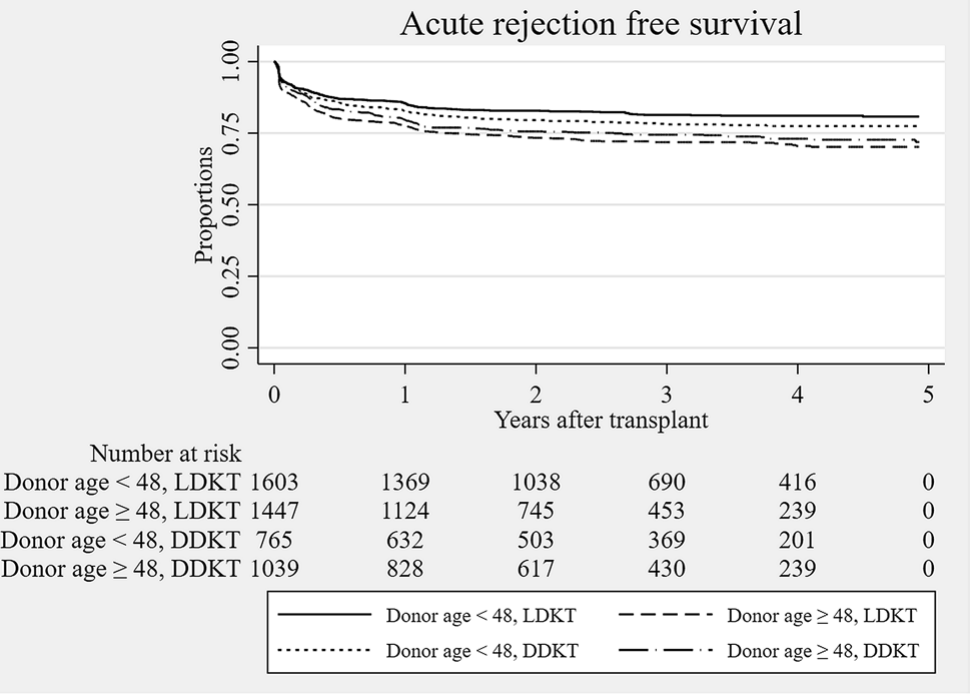
Acute rejection-free survival of kidney transplant recipients according to donor age and the types of organ donor. Donor age of 48 years old was selected as the optimal cutoff points among this study population. DDKT, deceased donor kidney transplantation; LDKT, living donor kidney transplantation.

**Figure 5:**
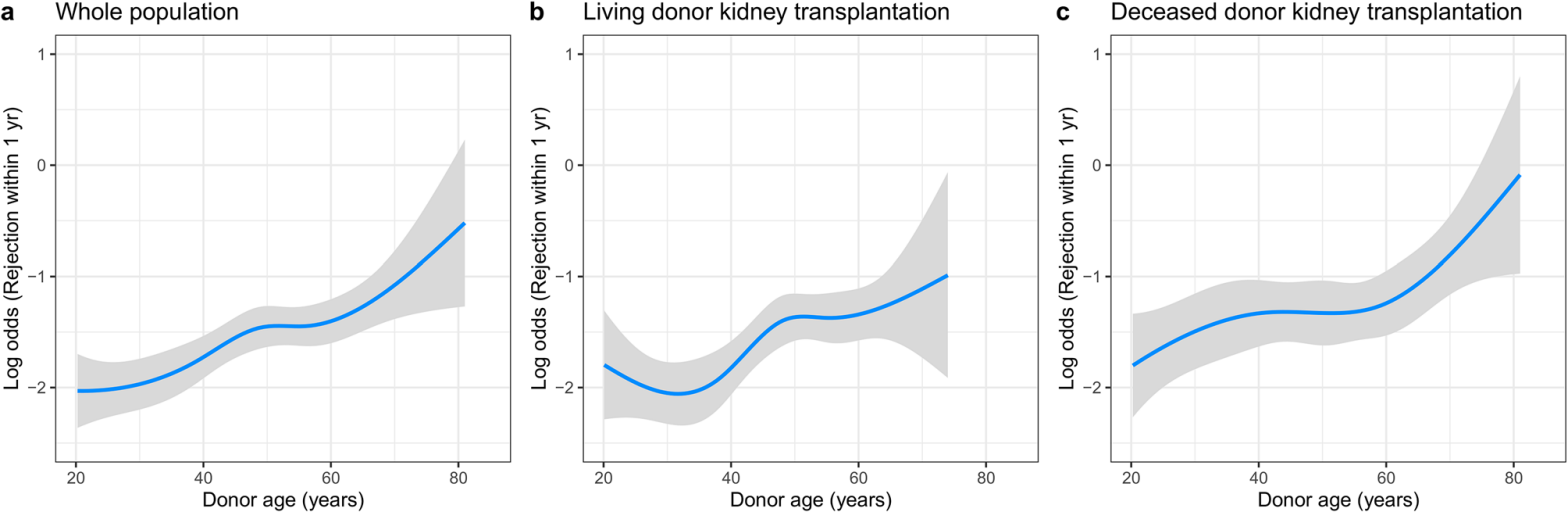
Visualization of log odds of acute rejection within 1 year according to different donor age (**a**) Visualization of log odds of acute rejection within 1 year according to different donor age in overall study population (**b**) Stratified log odds of acute rejection within 1 year according to different donor age in living donor kidney transplantation subpopulation (**c**) Stratified log odds of acute rejection within 1 year according to different donor age in deceased donor kidney transplantation subpopulation. Blue line indicates adjusted logarithm of odds of acute rejection within 1 year according to different donor age in each group. Each grey area indicates its 95% confidence interval. All graphs are the results of multivariable regression analyses which included donor age, HLA mismatch numbers, desensitization, recipient sex, recipient age, donor hypertension, recipient blood pressure, deceased donor, duration of renal replacement therapy, ever smoking in recipients.

Dominant predictors for death-censored 1-year graft survival were determined at the base of backward stepwise selection. (Supplementary Table 7) To the dominance analysis for post-transplant graft outcome, acute rejection and BK-virus-associated nephropathy (BKVAN) were included in the predictors list input to the backward stepwise selection. Acute rejection within 1 year followed by deceased donor, desensitization, and BK virus-associated nephropathy (BKVAN) were the dominant predictors to 1-year graft survival (Table 5). Dominant predictors for post-transplant 1-year graft eGFR were donor age, followed by acute rejection within 1 year, BKVAN within 1 year, recipient BMI and HLA mismatch numbers (Table 6). To derive important predictors to acute rejection, cross-validated LASSO was applied again, however, still LASSO resulted in all entered variables being selected at the optimum lambda. (Supplementary Table 8) Dominant factors for acute rejection within 1 year were determined at the base of backward stepwise selection. (Supplementary Table 9) Donor age, followed by HLA mismatch, desensitization, recipient sex, and recipient age were the high-ranked dominant predictors to acute rejection within 1 year (Table 7). Coefficient path plot of LASSO variable selection to the aforementioned outcomes are depicted in Supplementary contents (Supplementary Fig. 1). Because elderly donor showed reduced odds ratio, we checked the nonlinearity of donor age in acute rejection, and found that among deceased donor kidney transplantation recipients, young deceased donor under 20 years old showed significant non-linear elevated rejection risk. (Supplementary Fig. 2) To avoid this local non-linearity, we restricted the donor age above 19 years old, and found the same top priority of donor age to the acute rejection within 1 year in dominance analysis. In this subgroup analysis, donor age showed significant elevated odds ratio (Odds ratio 1.019 (95%. C.I 1.012–1.026, *p* < 0.001, Supplementary Table 10). (Fig. 3) In addition, we estimated the best cutoff points of donor age for the classification of acute rejection within 1 year by Liu’s methods. Donor age of 48 years old was the best cutoff for the classification of acute rejection. Fig. 4 is a Kaplan-Meier curve of post-transplant acute rejection free survival, which showed lower rejection-free survival of kidney transplant patients who received elderly donors more than 48 years old. However, increment of log odds of post-transplant 1 year rejection started from the late 20s of donor age and become steeper after 60 years old in the non-linear logistic regression analysis (Fig. 5).

**Table 5:** Selected predictors to 1 year death-censored graft loss and dominance.

Variables	Odds ratios (95% C.I.)	Beta	Standardized beta	*p*	Rank
Acute rejection within 1 year	6.169 (3.800–10.015)	1.867	0.724	< 0.001	1
Deceased donor	4.083 (2.179–7.650)	1.417	0.676	< 0.001	2
Desensitization	2.599 (1.333–5.067)	1.626	0.210	0.005	3
BKVAN within 1 year	5.052 (2.146–11.895)	0.655	0.236	< 0.001	4
Diabetes (recipients)	1.778 (1.074–2.946)	0.626	0.286	0.025	5
Donor hypertension	2.060 (1.194–3.553)	0.976	0.413	0.009	6
Systolic blood pressure (recipients), mmHg	0.989 (0.977–1.001)	− 0.008	− 0.173	0.066	7
Cancer (recipients)	1.995 (0.961–4.141)	0.684	0.170	0.064	8
Diabetes (donors)	0.335 (0.098–1.149)	− 1.461	− 0.323	0.082	9
Body mass index (recipients), kg/m^2^	1.053 (0.985–1.125)	0.048	0.171	0.133	10

BKVAN, BK virus associated nephropathy.

**Table 6:** Selected predictors to post-transplant 1 year estimated glomerular filtration rate and dominance.

Variables	Coefficients	95% C.I	*p*	Standardized beta	Rank
Donor age, yrs	− 0.597	− 0.646 to − 0.548	< 0.001	− 7.557	1
Acute rejection within 1 yr	− 10.522	− 12.065 to − 8.98	< 0.001	− 4.073	2
BKVAN within 1 yr	− 22.768	− 27.498 to − 18.038	< 0.001	− 2.856	3
Body mass index (recipients), kg/m^2^	− 0.461	− 0.636 to − 0.286	< 0.001	− 1.629	4
HLA mismatch numbers	− 0.344	− 0.689 to 0.001	0.051	− 0.606	5
Female donor	− 1.077	− 2.506 to 0.352	0.014	− 0.538	6
Ever smoking (donors)	2.156	0.703–3.610	0.004	− 1.027	7
Diabetes mellitus (donors)	− 2.202	− 5.032 to 0.627	0.127	− 0.472	8
Body mass index (donors), kg/m^2^	0.352	0.170–0.533	< 0.001	1.175	9
Deceased donor	− 1.822	− 3.442 to − 0.201	0.028	− 0.869	10
Systolic blood pressure (recipients), mmHg	0.054	0.024–0.083	< 0.001	1.106	11
Female recipients	0.571	− 0.691 to 1.833	0.375	0.281	12
Age (recipients)	− 0.013	− 0.067 to 0.040	0.632	− 0.150	13
Anti thymocyte globulin as induction agent	− 1.396	− 2.921 to 0.129	0.073	− 0.557	14
Duration of renal replacement therapy, months	0.011	− 0.001 to 0.022	0.067	0.662	15

BKVAN, BK virus associated nephropathy; HLA, human leukocyte antigen.

**Table 7:** Selected predictors to acute rejection within post-transplant 1 yr and dominance.

Variables	Odds ratios (95% C.I.)	Beta	Standardized beta	*p*	Rank
Donor age, yrs	0.988 (0.982–0.995)	0.020	0.250	< 0.001	1
HLA mismatch numbers	1.020 (1.013–1.027)	− 0.362	0.196	< 0.001	2
Desensitization	0.696 (0.591–0.820)	0.421	0.179	< 0.001	3
Female recipients	1.524 (1.272–1.825)	0.111	− 0.178	< 0.001	4
Recipient age, yrs	1.118 (1.07–1.168)	0.265	− 0.134	< 0.001	5
Hypertension (donors)	1.303 (1.058–1.605)	− 0.001	0.051	0.013	6
Diabetes mellitus (donors)	0.999 (0.997–1.000)	− 0.004	− 0.102	0.128	7
Systolic blood pressure (recipients), mmHg	0.997 (0.993–1.000)	− 0.169	− 0.072	0.062	8
Deceased donor	0.845 (0.702–1.017)	− 0.458	0.126	0.074	9
Duration of renal replacement therapy, months	0.633 (0.440–0.911)	0.140	− 0.071	0.014	10
Ever smoker (recipients)	1.150 (0.936–1.414)	0.020	− 0.072	0.184	11

HLA, human leukocyte antigen.

## Discussion

In the present study, we reported baseline characteristics and early outcomes based on the Korean Organ Transplantation Registry (KOTRY). In addition, we explored baseline predictors to early outcomes and reported dominant factors influencing patient and graft outcomes. Dominant factors for 1-year patient survival were found as predictors associated with recipient’s age or recipient’s comorbidities. Although infection was the most common cause of death, top dominant factor for 1-year patient survival was cardiovascular disease history. Aging might be intermediate process between infection as leading cause of death and cardiovascular history as leading predictor for patient 1 year mortality. For 1-year graft survival, dominant factors were predictors associated with immunologic risks and donor and recipient’s comorbidities. It was interesting to see that donor age was found to be the most dominant factor influencing graft function at 1 year, followed by post-transplant acute rejection and BKVAN.

When the KOTRY was launched, annual transplant numbers were 1,400. At the design stage of KOTRY, an annual enrollment of 1,200 cases was aimed at to cover more than 80% of total kidney transplants in South Korea. However, the recent rapid increase in kidney transplants has resulted in KOTRY covering about 50-60% of total kidney transplants in South Korea. Still, KOTRY projects compose the largest multi-center cohorts in this country. In KOTRY, clinical details that claim data cannot capture are important resources to future research. Another strength of KOTRY is its role as a biobank. Prospective sample collection will provide invaluable research resources.

The most common cause of ESRD in South Korea is diabetic nephropathy^24^, which is reflected in the high proportion of diabetes in KOTRY. A large proportion of glomerulonephritis as a cause of ESRD could represent the results of patient selection or accessibility to kidney transplantation. Another important feature of Korean kidney transplants is the high proportion of living donor kidney transplantation. We identified that among living donor kidney transplants, 24% were preemptive kidney transplants. Compared to the high proportion of preemptive transplants with living donor kidneys, a long waiting period among deceased donor kidney transplants is another feature of Korean kidney transplants. Aside from almost unanimous standard triple maintenance immunosuppressants, ATG induction was observed as a variation. The proportion of steroid withdrawal was 2% in this data. The most common cause of death was infection, followed by cardiovascular disease. Those causes of death are compatible with the predictors selected in the data-driven approach because recipient age and history of cardiovascular disease were selected as dominant predictors for 1-year mortality. The most common pre-transplant cardiovascular disease was ischemic heart disease in this study population. However, not only ischemic heart disease was significant predictors to post-transplant 1 year mortality, but other subcategories of cardiovascular disease were also significant predictors, which implies that holistic heart function itself is important for the early post-transplant mortality not limited to the presence or absence of coronary arterial occlusive disease.

Recent investigations of donor safety have concerned higher lifetime ESRD risk in young donors^25^. In terms of graft survival on the recipient side, it is interesting to see that selected predictors were donor characteristics such as donor age, donor hypertension, and donor diabetes. However, extension of this finding to long-term risk predictors needs cautious interpretation because non-modifiable donor factors could be exaggerated in early transplant outcomes. In terms of donor safety, marginal kidney function would also affect donors’ long-term outcomes; therefore, this data is evidence of the importance of proper donor selection.

It was interesting to see that donor age was the most dominant factor influencing acute rejection. Several publications pointed out the significance of donor age as a risk factor for acute rejection^26,27^; however, to the best of our knowledge, this is the first study to find that donor age is the most dominant factor influencing acute rejection in a quantitative comparison. Donor age has near-linear pattern of log odds increment, which might explain its dominancy in the regression-based dominance model. The best cutoff value which we can classify post-transplant acute rejection within 1 year with was 48 years old. However, it might be different from what we think as safety line of donor age, because the increment of log odds value is observed even in the late 20 s of donor age. In addition, the most profound increment of log odds of post-transplant rejection within 1 year from donor age was observed in the after 60 years old of donor age, especially among deceased donors. HLA incompatibility was the second most dominant predictor for acute rejection. This finding could be epidemiological evidence supporting the importance of passenger leukocyte and its memory, or the vulnerability of aged endothelial cells to ischemia reperfusion injury and damage-associated molecular pattern expression^28,29^. Desensitization was selected as an important predictor for acute rejection, which implies that although mitigation of immunological risk was performed by desensitization, residual risk still persisted. We anticipate that the details regarding desensitization will be investigated in future studies.

Dominant predictors to 1-year post-transplant recipient’s eGFR were donor age, acute rejection within 1 year, BKVAN within 1 year, and recipient BMI. We might interpret this as a mixture of kidney function and risk of rejection because donor age and body mass index can affect eGFR directly via muscle mass and intermediate outcomes including acute rejection, or that BKVAN directly represents the damaging process to the transplanted kidney. The importance of donor kidney-recipient weight gap was a well-known factor to post-transplant eGFR^30,31^. In this study, its importance to predicting post-transplant eGFR was high.

The limitations of the study are as follows. First, this project enrolled about 50% of total kidney transplant patients in South Korea. Informed consent was required; therefore, information bias might exist. For example, recipients with poor compliance could refuse study enrollment, and urgent transplants performed during weekends or late at night might not have been enrolled in this project. Second, dominance of predictors was based on variable selection in traditional stepwise regression, which is not completely independent as to the randomness of entering variables. We tried to overcome this limitation by applying regularized regression methods (LASSO), which were unsuccessful due to having a large number of cases compared to selected predictors. However, we think this quantitative comparison of the relative importance of variables is a significant contribution to the transplant field.

In conclusion, we presented clinical characteristics of patients enrolled in KOTRY during the past 5 years and investigated dominant predictors for early post-transplant outcomes by comparing relative contributions to the outcome prediction. The dominant predictors to recipient mortality within 1 year were deceased donor, recipient age, and recipient history of cardiovascular disease. The dominant predictors to death-censored graft loss within 1 year were deceased donor, desensitization, and donor hypertension. The dominant predictors of post-transplant 1-year recipient’s eGFR were donor age, acute rejection within 1 year, and BKVAN within 1 year. Finally, the dominant predictors to acute rejection within 1 year were donor age, HLA mismatches, and desensitization.

## Methods

### Study population

The Korean Organ Transplantation Registry (KOTRY) is a nationwide solid organ transplant cohort launched in 2014. The design and methods of KOTRY were described in detail in a previous report^11^. In brief, data on pretransplant evaluations, immunologic risks, induction and maintenance immunosuppressants, every kidney biopsy result, every treatment of acute rejection, graft function measured as eGFR, post-transplant cardiovascular events, post-transplant infection events, and the survival of patients and grafts were collected. As large-volume centers are participating in KOTRY, the numbers of organ transplantations performed in KOTRY-participating centers were 83% for kidney transplantation. However, because this nationwide cohort is based on patient’s informed consent and prospective follow up, KOTRY enrolls about 1,200 new kidney transplant cases per year, which reaches about 55% of annual total kidney transplantation in South Korea. Although some selection bias might exist for the patient enrollment, KOTRY data was shown to be compatible with the nationwide post-transplant hard outcome based on administrative claims^32^. For the details of post-transplantation outcome, KOTRY is the only source of Korean transplant recipient’s data in national scale. For this study, the dataset of patients who received kidney transplants from 2014 to 2018 was used. A total of 4,854 kidney transplant recipients were analyzed. Mean duration of follow-up was 36.4 ± 15.5 months.

### Study objective, design, covariables, and statistical approach

We tried to derive a best prediction model for post-transplant outcomes (patient survival, graft survival, post-transplant recipient’s eGFR, and acute rejection) from baseline (pre-transplant) covariables. For the variable selection, we applied various approaches depending on the availability of existing methods and the character of the variables. For continuous measures, the Furnival-Wilson leaps-and-bound algorithm determined by Akaike’s information criteria was used for variable selection^33,34^. For the time to event outcomes and binary outcomes, least absolute shrinkage and selection operator (LASSO) or backward stepwise selection were used. LASSO is a statistical methodology for variable selection and penalization. During the coefficient shrinkage of LASSO methodology, some coefficient goes to exactly zero value, which resulted in variable selection. For the LASSO, chosen optimum lambda values were one standard error apart from the lambda value of minimal partial likelihood deviance at the iterative cross-validation^35^. A total of 19 covariate candidates for prediction model construction were as follows: recipient age, donor age, recipient sex, donor sex, recipient’s history of diabetes, recipient’s history of cardiovascular disease, recipient’s history of cancer, pre-transplant systolic blood pressure of recipient, pre-transplant body mass index of recipient, donor’s diabetes history, donor’s hypertension history, waiting time to kidney transplant, pre-transplant body mass index of donor, deceased donor, total number of human leukocyte antigen (HLA) mismatches, desensitization, anti-thymocyte globulin (ATG) as an induction agent, and smoking status of donor and recipient. When all covariables were entered into the prediction model for the death-censored graft loss, c-statistic was 0.671, which was comparable to previous prediction studies^18^.

After a model was built, we applied dominance analysis to rank the relative importance of each selected variable to target outcome^36,37^. Because dominance analysis could be applied to the generalized linear model, we applied dominance analysis for continuous outcomes (post-transplant eGFR) in the form of linear regression, or for binary outcomes (patient survival at 1 year, graft survival status at 1 year, and acute rejection within 1 year) in the form of logistic regression. Because panel reactive antibody were tested in 62.1% of recipients, we did not include panel reactive antibody from the previous tests. We separately conducted the same process as a sensitivity analysis including panel reactive antibody as one of the predictors, which made total dataset reduced to 3,019 donor-recipients pairs. Panel reactive antibody was dropped during the backward variable selection and did not remain in the dominance analysis to rank the relative importance. Continuous data are presented as mean with standard deviation. Categorical data are presented as count with percent. Cox regression for time to event data was performed under the proportional hazard assumption. Splines were applied to formalize non-linearity in the statistical models. For the optimal cutoff value estimation, Liu’s method was used^38^. Statistical analyses were performed using Stata software (version 16; StataCorp LP, College Station, TX) and R (version 3.6.3; R Foundation, Vienna, Austria).

### Ethics approval and consent to participate

The study protocol was approved by the Seoul National University Hospital institutional review board (IRB No: H-1902-138-1014). Data analysis was done with de-identified datasets. Patient privacy was preserved in all instances, and the study methods complied with the tenets of the Declaration of Helsinki. All participants provided their written informed consent.

## Supplementary Information

Supplementary Figure 1.

Supplementary Figure 2.

Supplementary Information.

## Data Availability

The data that support the findings of this study are available from Korean Organ Transplantation Registry but restrictions apply to the availability of these data, which were used under license for the current study, and so are not publicly available. Data are however available from the authors upon reasonable request and with permission of Korean Organ Transplantation Registry.
