# Supplementary figures and images for: Dominant predictors of early post-transplant outcomes based on the Korean Organ Transplantation Registry (KOTRY)
Source: Sci Rep. 2022 May 24;12:8706. doi: 10.1038/s41598-022-12302-5 (PMC9130148; doi:10.1038/s41598-022-12302-5)

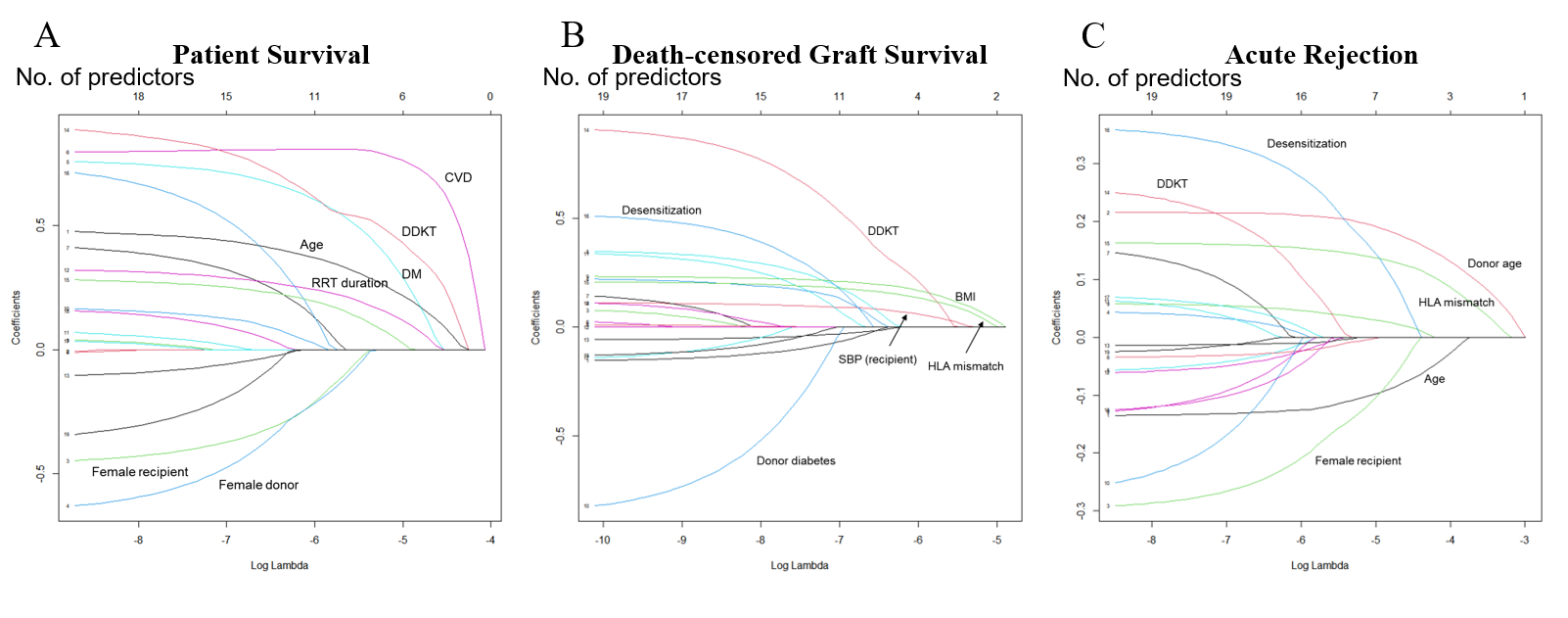

Supplement: Supplementary file 1 — Supplementary Figure 1. [file 41598_2022_12302_MOESM1_ESM.tif]

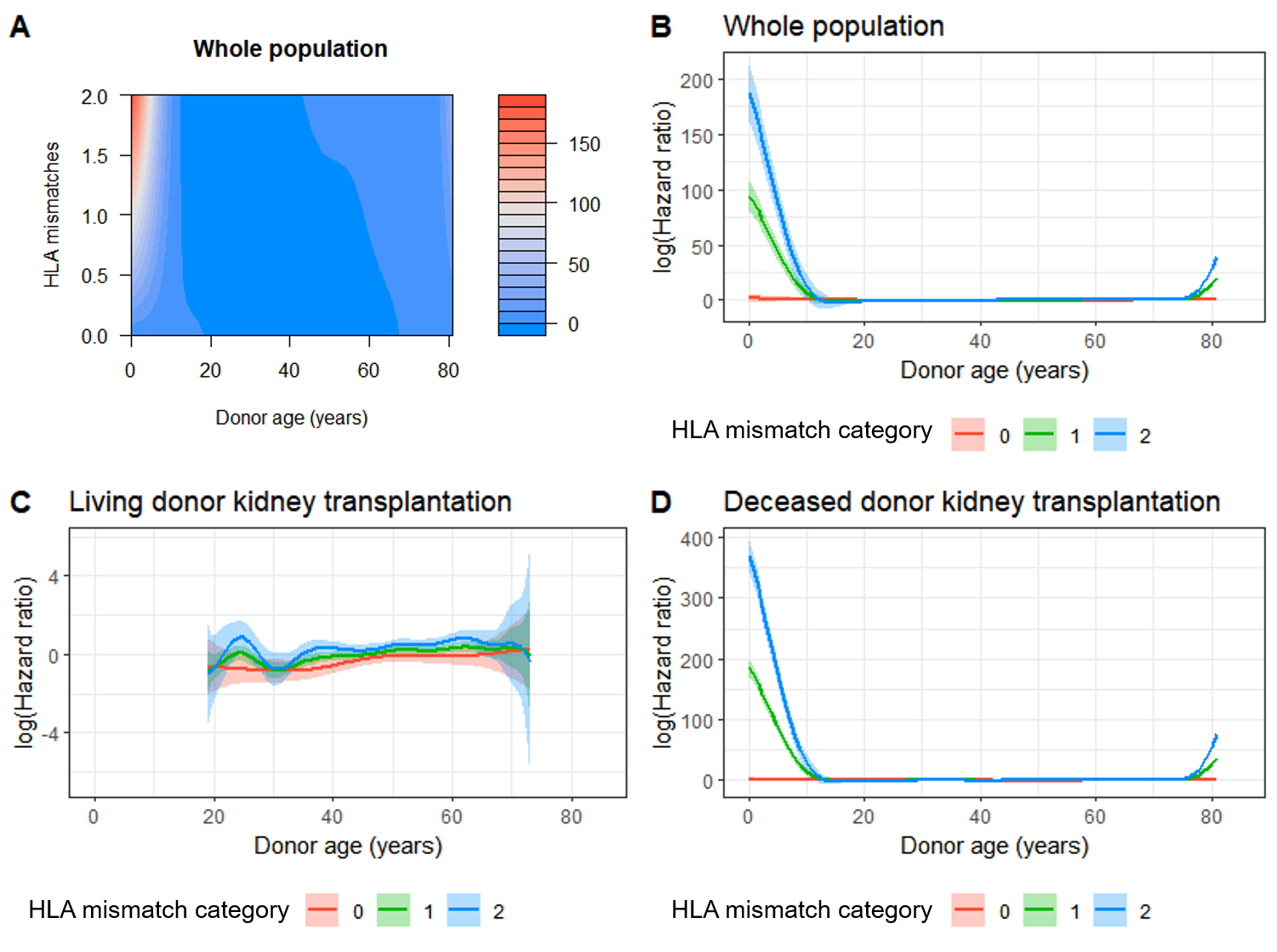

Supplement: Supplementary file 2 — Supplementary Figure 2. [file 41598_2022_12302_MOESM2_ESM.tif]
