# Supplementary material for: Dominant predictors of early post-transplant outcomes based on the Korean Organ Transplantation Registry (KOTRY)
Source: Sci Rep. 2022 May 24;12:8706. doi: 10.1038/s41598-022-12302-5 (PMC9130148; doi:10.1038/s41598-022-12302-5)
Supplement: Supplementary file 3 — Supplementary Information. [file 41598_2022_12302_MOESM3_ESM.pdf]

Supplemental Materials for

Dominant predictors of early post-transplant outcomes based on the Korean Organ Transplantation Registry (KOTRY)

Jong Cheol Jeong, Tai Yeon Koo, Han Ro, Dong Ryeol Lee, Dong Won Lee, Jieun Oh, Jayoun Kim, Dong-Wan Chae, Young Hoon Kim, Kyu Ha Huh, Jae Berm Park, Yeong Hoon Kim, Seungyeup Han, Soo Jin Na Choi, Sik Lee, Sang-Il Min, Jongwon Ha, Myoung Soo Kim, Curie Ahn, Jaeseok Yang* and the KOTRY study group

Supplemental Material File Listing

Supplementary Table 1

Supplementary Table 2

Supplementary Table 3

Supplementary Table 4

Supplementary Table 5

Supplementary Table 6

Supplementary Table 7

Supplementary Table 8

Supplementary Table 9

Supplementary Table 10

Supplementary Figure 1

Supplementary Figure 2

A full list of members and affiliation of Korean Organ Registry (KOTRY) consortium

Corresponding author information

Jaeseok Yang, MD, PhD

Transplantation Center, Seoul National University Hospital

Department of Surgery, Seoul National University Hospital

101 Daehak-no, Jongno-gu, Seoul 110-744, Republic of Korea

Telephone: +82-2-2072-4128

Fax: +82-2-2072-4129

E-mail: jcyjs@snu.ac.kr (JY)

Supplementary table 1. Causes of Death

Variables	Total (n=120)	Living (n=37)	Deceased (n=83)
Cardiovascular	12 (10.0%)	0 (0%)	12 (14.5%)
Infection	54 (45.0%)	18 (48.7%)	36 (43.4%)
Malignancy	9 (7.5%)	2 (5.4%)	7 (8.4%)
Sudden cardiac death	5 (4.2%)	4 (10.8%)	1 (1.2%)
Others	24 (20.0%)	7 (18.9%)	17 (20.5%)
Unknown	16 (13.3%)	6 (16.2%)	10 (12.1%)

Supplementary table 2. Causes of graft loss

Variables	Total (n=108)	Living (n=50)	Deceased (n=58)
Rejection	47 (43.5%)	24 (48%)	23 (39.7%)
BK virus nephropathy	6 (5.6%)	3 (6%)	3 (5.2%)
Glomerulonephritis	4 (3.7%)	0 (0%)	4 (6.9%)
Non-compliance	4 (3.7%)	3 (6%)	1 (1.7%)
Early surgical complication	3 (2.8%)	2 (4%)	1 (1.7%)
Primary graft failure	12 (11.1%)	5 (10%)	7 (12.1%)
Others	16 (14.8 %)	6 (12%)	10 (17.2%)
Unknown	16 (14.8%)	7 (14%)	9 (15.5%)

Supplementary table 3. Causes of Biopsies

Variables	Total (n=3,712)	Living (n=1,445)	Deceased (n=2,267)
Increased creatinine	1,382 (37.2%)	796 (35.1%)	586 (40.6%)
Increased proteinuria	74 (2.0%)	32 (1.4%)	42 (2.9%)
Protocol biopsy	2,174 (58.6%)	1,398 (61.7%)	776 (53.7%)
Others	82 (2.2%)	41 (1.8%)	41 (2.8%)

Supplementary table 4. Comparison of predictors to death of patient estimated by least absolute shrinkage and selection operator (LASSO) and multivariable Cox regression

Variables	Selective Inference by LASSO variable selection			Multivariable Cox regression		
	Coefficient	P-value	Post-selection interval	Coefficient	P-value	95% C.I.
Age (Recipients)	0.489	0.063	-0.044 - 0.707	0.043	<0.001	0.020 - 0.067
Age (Donors)	-0.021	0.842	-0.123 - 1.535	-0.003	0.669	-0.019 - 0.012
Sex (Recipients)	-0.464	0.067	-0.953 - 0.054	-0.466	0.064	-0.960 - 0.028
Sex (Donors)	-0.662	0.008	-1.128 - -0.222	-0.606	0.014	-1.091 - -0.12
DM history (Recipients)	0.770	0.008	0.283 - 1.145	0.732	0.001	0.291 - 1.174
CVD history (Recipients)	0.794	<0.001	0.421 - 1.162	0.787	<0.001	0.352 - 1.222
Cancer history (Recipients)	0.431	0.167	-0.340 - 0.945	0.416	0.181	-0.194 - 1.026
SBP (Recipients)	-0.019	0.851	-0.114 - 1.556	-0.001	0.821	-0.010 - 0.008
BMI (Recipients)	0.049	0.662	-0.727 - 0.207	0.019	0.550	-0.042 - 0.079
DM history (Donors)	0.179	0.584	-1.668 - 0.728	0.128	0.698	-0.518 - 0.774
HTN history (Donors)	0.089	0.731	-2.181 - 0.430	0.112	0.662	-0.391 - 0.616
Dialysis duration	0.327	<0.001	0.171 - 0.489	0.005	<0.001	0.002 - 0.008
BMI (Donors)	-0.115	0.260	-0.279 - 0.179	-0.029	0.336	-0.087 - 0.030

Deceased donor	0.913	0.022	0.199 - 1.822	0.940	0.002	0.334 - 1.545
HLA mismatch numbers	0.292	0.092	-0.079 - 0.472	0.173	0.007	0.048 - 0.298
Desensitization	0.756	0.013	0.219 - 1.447	0.758	0.012	0.165 - 1.351
ATG induction	0.045	0.844	-3.443 - 0.273	0.055	0.808	-0.388 - 0.498
Ever smoker (recipients)	0.170	0.472	-0.843 - 0.550	0.162	0.490	-0.297 - 0.621
Ever smoker (donors)	-0.376	0.102	-0.753 - 0.129	-0.377	0.099	-0.825 - 0.071

ATG, anti-thymocyte globulin; BMI, body mass index; CVD, Cardiovascular disease; DM, diabetes mellitus; HLA, human leukocyte antigen; HTN, hypertension; SBP, systolic blood pressure

Supplementary table 5. Selected predictors to patient death by stepwise backward selection

Variables	Hazard Ratio	95% C.I.	P
Age (recipient)	1.043	1.020 - 1.066	<0.001
Cardiovascular disease (recipient)	2.226	1.444 – 3.430	<0.001
Deceased donor	2.755	1.568 – 4.840	<0.001
RRT duration (months)	1.005	1.002 – 1.008	<0.001
Diabetes (recipient)	2.115	1.372 – 3.260	0.001
HLA mismatch numbers	1.186	1.050 – 1.339	0.006
Desensitization	2.174	1.207 – 3.917	0.017
Female recipient	0.590	0.372 – 0.935	0.025
Female donor	0.559	0.346 – 0.901	0.017
Ever-smoking (recipient)	0.706	0.453 – 1.102	0.125
Cancer (recipient)	1.495	0.814 – 2.745	0.195

HLA, human leukocyte antigen; RRT, renal replacement therapy

Supplementary table 6. Multivariable adjusted risk of cardiovascular disease subtypes

Variables	Hazard Ratio	95% C.I.	P
Arrhythmia history (recipient)	1.701	0.491 – 5.892	0.402
Ischemic heart disease history (recipient)	2.092	1.129 – 3.877	0.019
Heart failure history (recipient)	2.850	1.137 – 7.141	0.025
Valvular heart disease history (recipient)	5.395	1.108 – 26.269	0.037
Other heart disease history (recipient)	6.746	1.488 – 30.587	0.013
Deceased donor	3.213	1.560 – 6.618	0.002
Age (recipient)	1.044	1.015 - 1.073	0.003
RRT duration (months)	1.006	1.002 – 1.009	0.002
Diabetes (recipients)	1.607	0.925 – 2.790	0.092
Diabetes (donors)	1.573	0.756 – 3.271	0.225
HLA mismatch numbers	1.104	0.952 – 1.281	0.192
Body mass index (recipients), kg/m^2	1.056	0.981 – 1.137	0.149
Body mass index (donors), kg/m^2	0.953	0.888 – 1.022	0.177
Desensitization	2.665	1.260 – 5.638	0.010
Female recipient	0.711	0.410 – 1.231	0.223

HLA, human leukocyte antigen; RRT, renal replacement therapy

Supplementary table 7. Selected predictors to death-censored graft loss by stepwise backward selection

Variables	Hazard Ratio	95% C.I.	P
Deceased donor	2.305	1.508 - 3.523	<0.001
Body mass index (recipient)	1.058	1.009 - 1.11	0.021
HLA mismatch numbers	1.094	0.987 - 1.213	0.087
Hypertension (donor)	1.452	0.948 - 2.224	0.086
Desensitization	1.465	0.917 - 2.339	0.110
Female donor	1.316	0.921 - 1.882	0.132
Diabetes (donor)	0.477	0.202 - 1.129	0.092
Diabetes (recipient)	1.326	0.921 - 1.909	0.129
Acute rejection within 1 yr	4.285	3.038 - 6.044	<0.001
BKVAN within 1 yr	3.779	1.973 - 7.24	<0.001

BKVAN, BK virus associated nephropathy; HLA, human leukocyte antigen

Supplementary table 8. Comparison of predictors to acute rejection estimated by least absolute shrinkage and selection operator (LASSO) and multivariable Cox regression

Variables	Selective Inference by LASSO variable selection			Multivariable Cox regression		
	Coefficient	P-value	Post-selection interval	Coefficient	P-value	95% C.I.
Age (Recipients)	-0.136	<0.001	-0.192 - -0.081	-0.012	<0.001	-0.018 - -0.006
Age (Donors)	0.216	<0.001	0.158 - 0.275	-0.290	<0.001	0.012 - 0.023
Sex (Recipients)	-0.300	<0.001	-0.419 - -0.18	0.050	<0.001	-0.432 - -0.149
Sex (Donors)	0.046	0.446	-0.329 - 0.347	-0.048	0.510	-0.098 - 0.197
DM history (Recipients)	-0.060	0.418	-0.179 - 0.227	-0.138	0.514	-0.194 - 0.097
CVD history (Recipients)	-0.138	0.207	-0.318 - 0.151	0.145	0.205	-0.351 - 0.075
Cancer history (Recipients)	0.16	0.183	-0.144 - 0.357	-0.002	0.230	-0.091 - 0.380
SBP (Recipients)	-0.035	0.272	-0.087 - 0.059	0.017	0.249	-0.005 - 0.001
BMI (Recipients)	0.059	0.064	-0.006 - 0.111	-0.277	0.051	0.001 - 0.035
DM history (Donors)	-0.277	0.067	-0.525 - 0.032	0.080	0.062	-0.568 - 0.014
HTN history (Donors)	0.07	0.425	-0.274 - 0.209	-0.001	0.360	-0.091 - 0.251
Dialysis duration	-0.065	0.097	-0.129 - 0.02	-0.005	0.086	-0.002 – 0.001
BMI (Donors)	-0.014	0.657	-0.06 - 0.207	0.095	0.003	0.091 - 0.453

Deceased donor	0.264	0.004	0.105 - 0.422	0.371	<0.001	0.059 - 0.132
HLA mismatch numbers	0.164	<0.001	0.109 - 0.218	0.066	<0.001	0.220 - 0.521
Desensitization	0.366	<0.001	0.239 - 0.493	-0.134	0.389	-0.085 - 0.217
ATG induction	0.073	0.342	-0.186 - 0.199	-0.017	0.086	-0.288 - 0.019
Ever smoker (recipients)	-0.133	0.092	-0.262 - 0.036	0.001	0.829	-0.167 - 0.134
Ever smoker (donors)	-0.028	0.688	-0.197 - 0.615	-0.012	<0.001	-0.018 - -0.006

ATG, anti-thymocyte globulin; BMI, body mass index; CVD, Cardiovascular disease; DM, diabetes mellitus; HLA, human leukocyte antigen; HTN, hypertension; SBP, systolic blood pressure

Supplementary table 9. Selected predictors to acute rejection by stepwise backward selection

Variables	Hazard Ratio	95% C.I.	P
Age (Recipient)	0.988	0.983 – 0.993	<0.001
Age (Donors)	1.018	1.013 – 1.023	<0.001
Female recipient	0.748	0.652 – 0.858	<0.001
Deceased donor	1.307	1.106 – 1.545	0.002
Ever smoker (recipient)	0.867	1.106 – 1.545	0.002
Cardiovascular disease	0.865	0.701 – 1.068	0.177
Desensitization	1.464	1.262 – 1.699	<0.001
RRT duration	0.999	0.998 – 1.000	0.113
HLA mismatch numbers	1.101	1.062 – 1.141	<0.001
Body mass index (Recipients)	1.016	0.999 – 1.034	0.064
DM history (Donors)	0.732	0.575 – 1.017	0.065

DM, diabetes mellitus; HLA, human leukocyte antigen; RRT, renal replacement therapy

Supplementary table 10. Selected predictors to acute rejection within post-transplant 1yr and dominance in the subpopulation whose donor age is over 19 years old.

Variables	Odds Ratios (95% C.I.)	Beta	Standardized Beta	P	Rank
Donor age, yrs	1.019 (1.012 - 1.026)	0.018	0.223	<0.001	1
HLA mismatch numbers	1.121 (1.071 - 1.173)	0.114	0.201	<0.001	2
Desensitization	1.541 (1.277 - 1.859)	0.432	0.184	<0.001	3
Female recipients	0.685 (0.577 - 0.812)	-0.379	-0.186	<0.001	4
Recipient age, yrs	0.989 (0.983 - 0.996)	-0.011	-0.125	0.002	5
Deceased donor	1.398 (1.128 - 1.733)	0.335	0.159	0.002	6
Diabetes mellitus (donors)	0.996 (0.992 – 1.000)	-0.352	-0.076	0.058	7
Duration of renal replacement therapy, months	0.703 (0.489 - 1.010)	-0.001	-0.079	0.056	8
Systolic blood pressure (recipients), mmHg	0.999 (0.997 – 1.000)	-0.004	-0.079	0.101	9
Ever smoker (recipients)	0.841 (0.694 - 1.020)	-0.173	-0.074	0.078	10

HLA, human leukocyte antigen

Supplementary Figure 1. Coefficient path plot of the prediction model by LASSO to various post-transplant outcomes. (A) Coefficient path plot to patient survival (B) Coefficient path plot to death-censored graft survival (C) Coefficient path plot to acute rejection

Upper x-axis indicates included numbers of predictors in regularized LASSO models at certain log lambda values. Numeric labels indicate each predictors as follows: 1, recipient age; 2, donor age; 3, female recipient; 4, female donor; 5, diabetic recipient; 6, history of cardiovascular disease in recipient; 7, history of cancer in recipient; 8, systolic blood pressure in recipient; 9, body mass index in recipient; 10, donor diabetes; 11, donor hypertension; 12, duration of renal replacement therapy; 13, systolic blood pressure in donor; 14, body mass index in donor; 15, deceased donor; 16, HLA mismatch numbers; 17, desensitization; 18, anti-thymocyte globulin induction; 19, ever-smoker (recipient); 20, ever-smoker (donor)

Supplementary Figure 2. Depiction of non-linearity of donor age to post-transplantation acute rejection (A) Two dimensional visualization of log odds of rejection within 1 year according to donor age and HLA mismatch numbers in overall study population (B) Log odds of rejection within 1 year according to donor age and HLA mismatch numbers in overall study population (C) Stratified log odds of rejection within 1 year according to donor age and HLA mismatch numbers in living donor kidney transplantation subpopulation (D) Stratified log odds of rejection within 1 year according to donor age and HLA mismatch numbers in deceased donor kidney transplantation subpopulation

Red line indicates logarithm of hazard ratio of donor age in HLA full match group. Each colored area indicates its 95% confidence interval. Green line indicates logarithm of hazard ratio of donor age in moderate HLA mismatch group (mismatch numbers 1 to 4). Blue line indicates logarithm of hazard ratio of donor age in high HLA mismatch group (mismatch numbers 5 to 6). All graphs are the results of multivariable regression analyses which included donor age, HLA mismatch numbers, desensitization, recipient sex, recipient age, donor hypertension, recipient blood pressure, deceased donor, duration of renal replacement therapy, ever smoking in recipients.

CONSORTIUM

KOTRY study group

Curie Ahn[8,18], Myoung Soo Kim[10], Jaeseok Yang[19], Jin Min Kong[20], Oh Jung Kwon[21], Deok Gie Kim[22], Cheol Woong Jung[23], Yeong Hoon Kim[12], Joong Kyung Kim[24], Chan-Duck Kim[25], Ji Won Min[26], Sung Kwang Park[15], Yeon Ho Park[27], Jae Berm Park[11], Jung Hwan Park[28], Jong-Won Park[29], Tae Hyun Ban[30], Sang Heon Song[5], Seung Hwan Song[31], Ho Sik Shin[32], Chul Woo Yang[33], Hye Eun Yoon[34], Kang Wook Lee[35], Dong Ryeol Lee[4], Dong Won Lee[5], Sam Yeol Lee[36], Sang-Ho Lee[37], Su Hyung Lee[38], Yu Ho Lee[39], Jung Pyo Lee[40], Jeong-Hoon Lee[41], Jin Seok Jeon[42], Heungman Jun[43], Kyunghwan Jeong[44], Ku Yong Chung[45], Hong Rae Cho[46], Ju Man Ki[47], Dong-Wan Chae[1], Soo Jin Na Choi[14], Sung Shin[9], Seungyeup Han[13], Kyu Ha Huh[10]

[20]Department of Nephrology, BHS Hanseo Hospital

[21]Department of Surgery, College of Medicine, Han Yang University

[22]Department of Surgery, Yonsei University Wonju College of Medicine, Wonju Severance Christian Hospital

[23]Department of Surgery, Korea University Anam Hospital

[24]Department of Internal Medicine, Bongseng Memorial Hospital

[25]Department of Internal Medicine, School of Medicine, Kyungpook National University Hospital

[26]Division of Nephrology, Department of Internal Medicine, Bucheon St. Mary's Hospital

[27]Department of Surgery, Gil Medical Center, Gachon University College of Medicine

[28]Konkuk University School of Medicine, Department of Nephrology

[29]Department of Nephrology, Yeungnam University Hospital

[30]Division of Nephrology, Department of Internal Medicine, Eunpyeong St. Mary's hospital

[31]Department of Surgery, Ewha Womans University Seoul Hospital

[32]Kosin University College of Medicine, Department of Internal Medicine, Division of Nephrology

[33]Division of Nephrology, Department of Internal Medicine, Seoul St. Mary's hospital

[34]Department of Internal Medicine, Incheon St. Mary’s Hospital, College of Medicine, The Catholic University of Korea College of Medicine

[35]Department of Nephrology, Chungnam National University Hospital

[36]Department of Surgery, Kangdong Sacred Heart Hospital, Hallym University College of Medicine

[37]Department of Nephrology, Kyung Hee University Hospital at Gangdong

[38]Department of Surgery, Ajou University School of Medicine

[39]Division of Nephrology, Department of Internal Medicine, CHA Bundang Medical Center, CHA University, Seongnam, Korea

[40]Department of Nephrology, SMG-SNU Boramae Medical Center

[41]Department of Surgery, Myongji Hospital

[42]Department of Internal Medicine, Soonchunhyang University Seoul Hospital

[43]Department of Surgery, Inje University Ilsan Paik Hospital

[44]Department of Internal Medicine, Kyung Hee University College of Medicine

[45]Department of Surgery, Ewha Womans University Mokdong Hospital

[46]Department of Surgery, Ulsan University Hospital

[47]Department of Surgery, Gangnam Severance Hospital, Yonsei University College of Medicine
